# Multifunctional microfluidic chip for optical nanoprobe based RNA detection – application to *Chronic Myeloid Leukemia*

**DOI:** 10.1038/s41598-017-18725-9

**Published:** 2018-01-10

**Authors:** Pedro Urbano Alves, Raquel Vinhas, Alexandra R. Fernandes, Semra Zuhal Birol, Levent Trabzon, Iwona Bernacka-Wojcik, Rui Igreja, Paulo Lopes, Pedro Viana Baptista, Hugo Águas, Elvira Fortunato, Rodrigo Martins

**Affiliations:** 1 CENIMAT/I3N, Departamento de Ciência dos Materiais, Faculdade de Ciências e Tecnologia, Universidade Nova de Lisboa and CEMOP/UNINOVA, Campus de Caparica, 2829-516 Caparica Portugal; 2 UCIBIO, Departamento de Ciências da Vida, Faculdade de Ciências e Tecnologia, Universidade Nova de Lisboa, Campus de Caparica, 2829-516 Caparica Portugal; 3 MEMS, Department of Nanoscience and Nanoengineering, Istanbul Technical University, Ayazaga Campus, 34469 Maslak Turkey; 4 Laboratory of Organic Electronics, Department of Science and Technology, Linköping University, SE-601 74 Norrköping, Sweden; 5 Department of Physics and IEETA (Institute of Electronics and Informatics Engineering of Aveiro), Campus Santiago, University of Aveiro, Aveiro, 3810-193 Portugal

## Abstract

Many diseases have their treatment options narrowed and end up being fatal if detected during later stages. As a consequence, point-of-care devices have an increasing importance for routine screening applications in the health sector due to their portability, fast analyses and decreased cost. For that purpose, a multifunctional chip was developed and tested using gold nanoprobes to perform RNA optical detection inside a microfluidic chip without the need of molecular amplification steps. As a proof-of-concept, this device was used for the rapid detection of chronic myeloid leukemia, a hemato-oncological disease that would benefit from early stage diagnostics and screening tests. The chip passively mixed target RNA from samples, gold nanoprobes and saline solution to infer a result from their final colorimetric properties. An optical fiber network was used to evaluate its transmitted spectra inside the chip. Trials provided accurate output results within 3 min, yielding signal-to-noise ratios up to 9 dB. When compared to actual state-of-art screening techniques of chronic myeloid leukemia, these results were, at microscale, at least 10 times faster than the reported detection methods for chronic myeloid leukemia. Concerning point-of-care applications, this work paves the way for other new and more complex versions of optical based genosensors.

## Introduction

Nanoparticle-based bioanalyte detection has been at the forefront of miniaturized systems for molecular diagnostics at point of care (POC) testing. In particular, gold nanoparticles (AuNPs) have been revolutionizing the molecular field of optical analysis due to their good stability, visible color change between aggregate and non-aggregate state, and affinity with biomolecules^1–4^. The first reports on molecular detection of DNA based on the cross-linking of AuNPs with color change^5^, have paved the way for several different approaches to circumvent limitations associated to current Polymerase Chain Reaction (PCR) based methods. Baptista *et al*. have been developing a non-cross-linking colorimetric method based on the differential aggregation of oligonucleotide functionalized AuNPs, and hence applied it to the detection of DNA (pathogens, human genomic, etc.). More interestingly, this system has also been used for the detection of RNA since it enables screening and characterization of transcripts, allowing gene expression studies and genetic disease studies^6,7^. Several of these systems have been further incorporated into microfluidics platforms for enhanced detection capabilities^8–13^. Microfluidic technology provides the means for miniaturization of chemical and biochemical analysis that may easily be made portable^14–16^. They bring several advantages when compared to standard apparatus, such as the significant decrease in volumes of reagents and samples, faster operation and reaction times, decreased analysis time and decreased costs^17–19^. These characteristics can be synergistically and simultaneously combined on microfluidic platforms^20,21^, making microfluidic devices promising for research purposes^22^, for high value applications in the medical and pharmaceutical industries^23^, and ideal for POC^24^. Optics is an effective mean of transduction that can be synergistically paired with microfluidics to design highly compact and integrated devices^25^. Recent works, which use different types of nanoparticles to either provide^26^ or enhance^27^ optical output signals, have reported significant progress towards POC applications. Lately, microfluidics has also burgeoned into paper-based biosensors^28^, as they provide extremely cheap cellulosic material, compatible with many chemical/ biochemical/ medical applications and allow the transportation of liquids without external forces due to capillary forces^29,30^. However, this technology still faces some limitations^31^, such as the sample retention within channels and sample evaporation during transport, which result in low efficiency of sample-delivery inside the device (usually less than 50%). The limit of detection (LOD) on paper microfluidics is also poorer when compared to the conventional microfluidics due to its incapacity of analyzing samples at low concentrations^31^. Due to these constrains, and despite requiring more expensive equipment, conventional microfluidics is chosen regularly for POC applications^32^.

**Figure 1:**
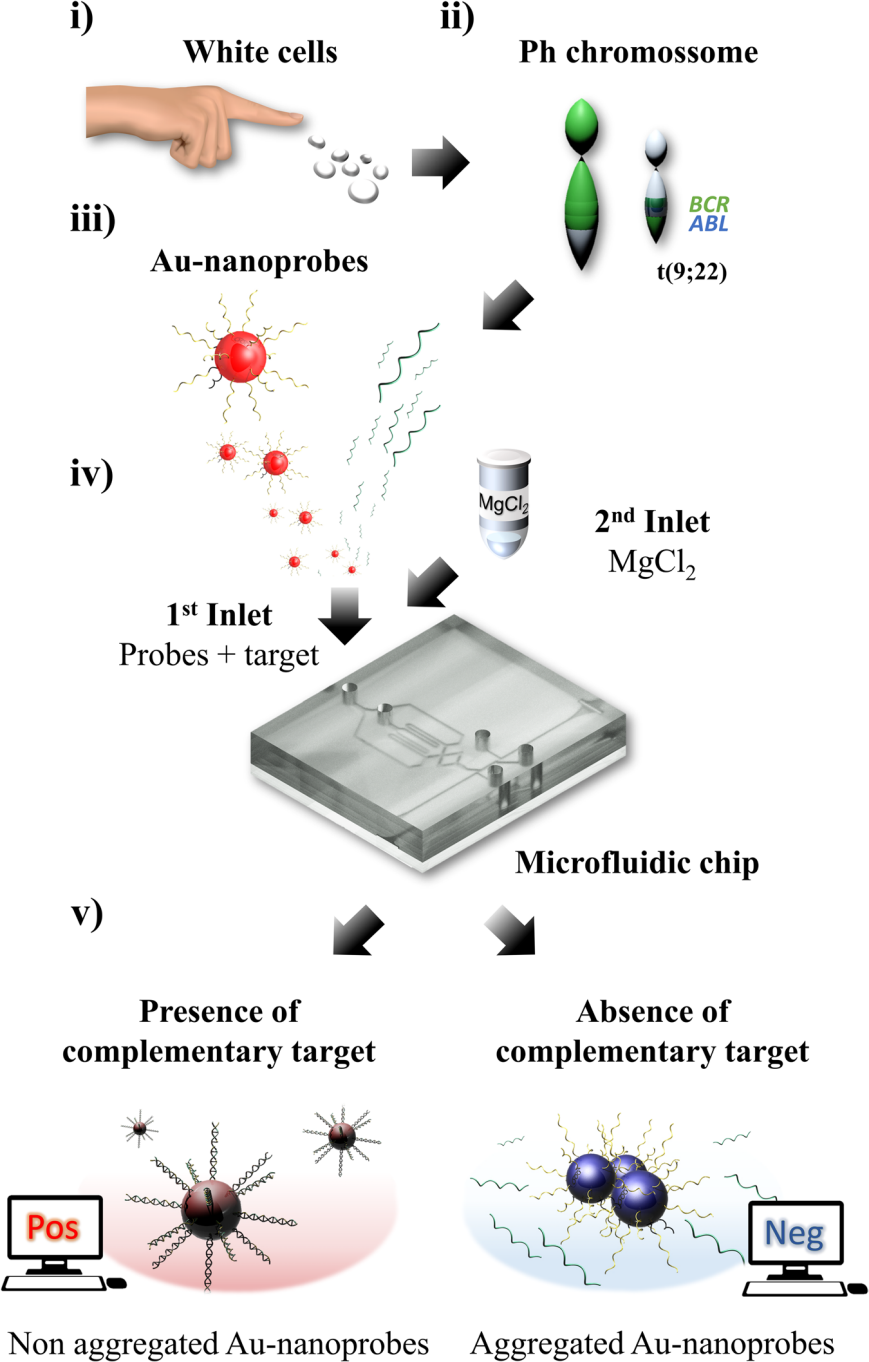
Concept of this lab-on-chip as a POC application: (**i**) White cells extracted from a small blood sample are collected to analyze gene expression; (**ii**) This approach aims to diagnose chronic myeloid leukemia using its genetic marker, *BCR-ABL1* fusion transcripts, present in the cells; (**iii**) Total RNA extracted is then mixed with Au-nanoprobes and heated to promote hybridization. Note that Au-nanoprobes are functionalized with *BCR-ABL1* complementary sequences; (**iv**) The resulting solution and a salt solution are infused on the two microfluidic chip inlets; (**v**) Thorough mixing and optical detection of these components is performed inside the microfluidic chip. If the patient expresses *BCR-ABL1* transcripts complementary to the oligonucleotide sequence of Au-nanoprobes, their hybridization will cause the final solution to remain red (positive match) in the presence of salt. Otherwise, the non-hybridized Au-nanoprobes will aggregate and cause the final solution to turn blue (negative match) in the presence of salt. Output results described in this step are displayed on the computer within 3 min.

This work describes in detail a novel and low-cost conventional Polydimethylsiloxane (PDMS) microfluidic chip which combines a short-path length micromixer and an optical circuit to perform colorimetric detection (Fig. 1). To demonstrate the impact of this microfluidic chip for molecular diagnostics, gold-nanoprobe (Au-nanoprobe) assays were applied in the detection of *BCR-ABL1* fusion transcript (RNA), which is the molecular hallmark of chronic myeloid leukemia (CML).

CML is originated from the Philadelphia chromosome (Ph) - a reciprocal translocation of the long arms of chromosomes 9 and 22, t(9;22). This aberrant chromosome is found in more than 90% patients with CML, 15–25% of patients with acute lymphoblastic leukemia (ALL) and 1% of newly diagnosed adults with acute myeloid leukemia^33–35^. The Philadelphia chromosome results from the fusion of two genes, *BCR* and *ABL1*. The chimeric gene leads to the translation of a constitutively active protein tyrosine kinase. Although several transcript isoforms have been reported, for the purpose of this study the Au-nanoprobe was designed towards the most frequent variant, e14a2, accounting for more than 55% of CML patients^36,37^. The high treatment success rate of the disease relies not only on drug efficacy, but also on fast, accurate and early diagnostic tools^38^. Due to the scarcity of such tools, intense research has been devoted to the development of new methodologies for CML screening and management.

Following design, fabrication and characterization of the chip, three case studies with increasing complexity were performed and analyzed in the present report: first, Au-nanoprobes were used with and without salt to make the proof of concept; second, BCR-ABL1 synthetic oligonucleotides were combined with Au-nanoprobes to evaluate the mixing and cross-linking behavior under mixing and optical detection inside the microchannel; and third, the system performance was assessed using total RNA extracted from a CML cell line, which is the surrogate model for the disease, thus mimicking real clinical sample screening. These results on-chip, obtained without requiring any amplification steps, were faster than the standard laboratory operation process at macroscale^7^ and unprecedented at microscale even when compared with other similar and up to date methods^39,40^. They have also provided a cheaper solution to methods that recur to fluorescence^41,42^, and a simpler fabrication and cost efficiency when compared to other techniques^43,44^ without any significant losses in sensitivity. These devices are suitable for the fast screenings required in medical care and POC, and key to unlock new and more complex versions of optical based genosensors, either to diagnose CML or other similar diseases.

## Results and Discussion

### Microfluidic chip design

**Figure 2:**
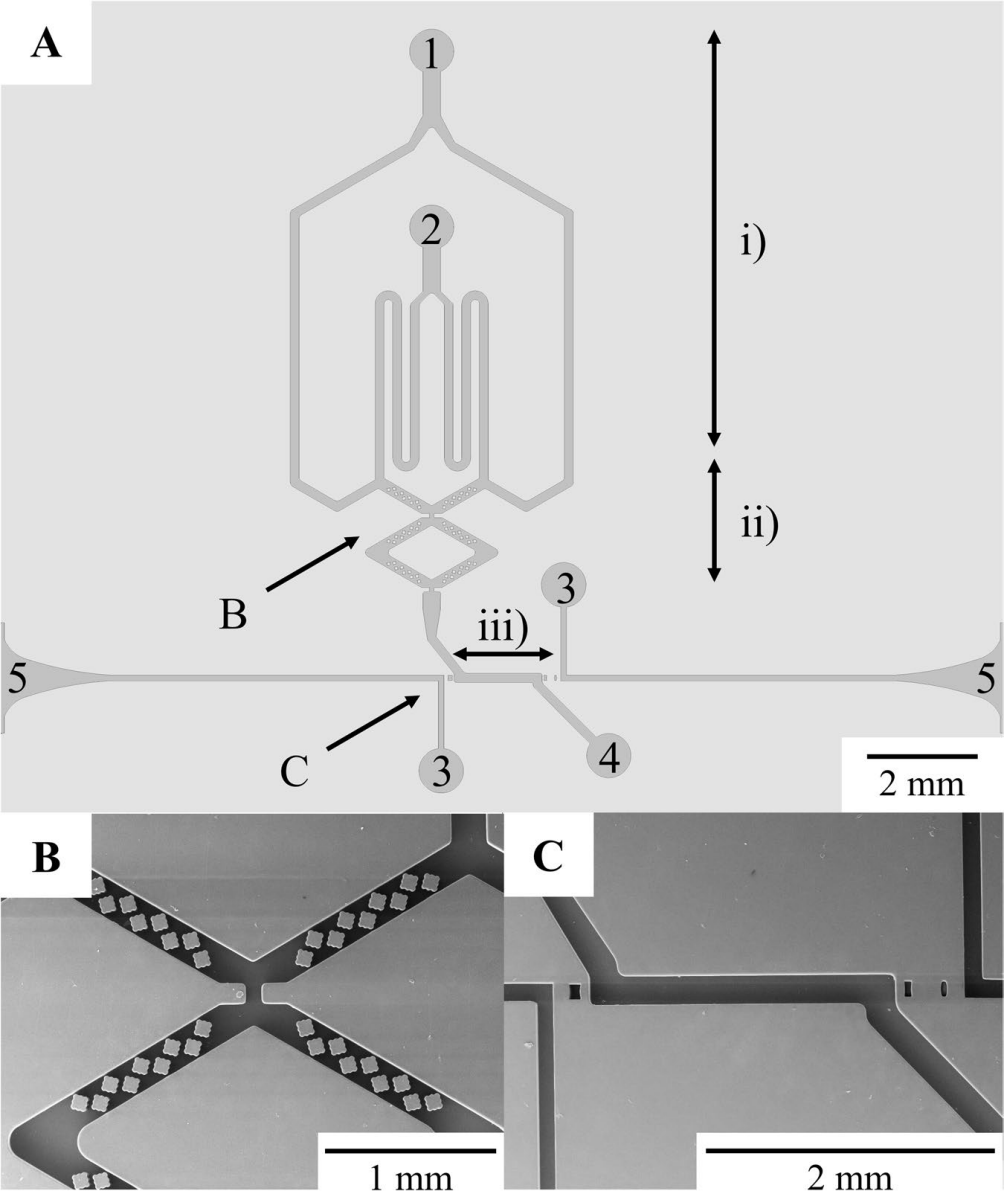
(**A**) Microfluidic chip design: (i) infusion section; (ii) mixing section; (iii) optical detection section; 1 – Target DNA/RNA and Au-nanoprobes solution inlet; 2 – Salt (MgCl_2_) inlet; 3 – Optical fiber cleaning channel; 4 – Microchannel outlet; 5 – optical fiber insertion cavity. (**B**) Detailed Scanning Electron Microscopy (SEM) picture of the mixing region. The mixing region takes advantage of 1.5 rhombi, each with 36 diamond shaped obstacles and 2 throttles to perform efficient passive mixing. (**C**) Detailed SEM picture of the detection region. A collimating lens is used between the first optical fiber groove and microchannel to align the incoming light onto the channel. Likewise, two focusing lenses are used between microchannel and the second optical fiber groove to focus the outcoming light onto the output optical fiber.

The microfluidic chip layout was conceived by combining 3 distinct sections (Fig. 2A). The first section allowed infused solutions to reach the common channel simultaneously (Fig. 2A - i). This synchronization allows a more efficient utilization since no solution will be left unmixed.

The second component of the chip had a planar micromixer incorporated to perform mixing between solutions (Fig. 2A - ii). This micromixer was chosen over active and 3D micromixers^45,46^ due to its cost efficiency and easy integration with other components of the microchannel, thus allowing low cost fabrication since it only requires a single layer of photolithography to yield the full design. Among passive planar micromixers, rhombic micromixers with obstacles^47^ are suitable for this application as they fulfill the mixing requirements over a wide range of Reynold numbers and for short mixing channels (2.5 mm), and thus they were implemented in the described device. The integrated mixer has one and a half rhombi, and each edge has a total of nine diamond shaped obstacles to disturb the laminar flow and force mixing between solutions (Fig. 2B). Each rhombus is also separated by throttles that seek to increase mixing efficiency even further by creating pressure gradients with an abrupt variation on the channel’s width. These variations tend to generate vortexes, and thus, to enhance mixing.

The third and last section of the chip comprises the optical detection unit, where the nucleic acid colorimetric analysis takes place (Fig. 2A - iii). This screening takes advantage of an extended optical path length design inside the chip^13^, which uses optical fibers to convey light between source and microfluidic chip, and between microfluidic chip and photodiode. Air lenses were incorporated at the entrance and exit, between the optical fiber grooves and the detection channel, to minimize signal losses^12^; the former collimates light coming out of the fiber tip and illuminates the microchannel content uniformly, and the latter focuses the outgoing light into the core of the output optical fiber. Two optical grooves were also integrated in the chip design to enable approximation and alignment between optical fibers and channel (this is where the optical fiber circuit crosses paths with the microfluidic channel). Built-in cleaning channels were added to allow an easy removal of dust and particles before the optical fiber insertion (Fig. 2C). Green (530 nm) and red (625 nm) light emitting diodes, detector and electrical setup were kept fixed, while disposable microfluidic chips and attached optical fibers can be replaced with ease for a cleaner usage. LED wavelengths were chosen according to the absorption spectra of aggregated and non-aggregated Au-nanoprobes^48,49^. The low price, biocompatibility and optical properties of PDMS made this silicon rubber a common choice to fabricate the microfluidic chips by replica molding^50,51^. Full details on the chip design can be seen in Supplementary Information – Fig. S1.

### Optical setup

An optoelectronic setup was assembled to integrate the microfabricated chips and to perform optical analysis. It included two wavelength light sources (625 nm LED; 530 nm LED) which emit light through an optical fiber. This optical fiber crosses the microfluidic channel and interacts with the infused solution. A syringe pump was used to infuse solutions inside the chip. A photodetector transduced out coming light to a current signal, which was amplified by an operation amplifier with a feedback network (capacitor: 1.5 nF; resistance: 20 MΩ), and converted to a voltage signal. This analog signal was then converted to digital and acquired by the computer. Full details on the setup can be found in Supplementary Information – Fig. S2.

The detection response (*R*_s_) was defined as the ratio of the digital output acquired by the computer of the red LED (dominant wavelength: 625 nm) and green LED (dominant wavelength: 530 nm) on a screened sample:

1 $${R}_{s}=\frac{Sample\,{V}_{out}\,(625\,nm)}{Sample\,{V}_{out}\,(530\,nm)}/\frac{Baseline\,{V}_{out}\,(625\,nm)}{Baseline\,{V}_{out}\,(530\,nm)}$$

This ratio was normalized to a baseline sample ratio, 10 mM phosphate buffer pH 8 solution, as it eliminates the intrinsic optical properties that vary from chip to chip or with time and usage^12,13^. Baseline data was extracted prior to and after each assay in order to confirm the system reliability.

### Colorimetric analysis

Hybridization of Au-nanoprobes to targets was performed in 10 mM phosphate buffer pH 8, followed by a 5 min denaturation at 95 °C. Samples were then cooled down to room temperature before injection into the chip. The microfluidic chip was rinsed with isopropanol to increase the microchannel hydrophilicity and to avoid formation of bubbles that could otherwise affect optical readings and increase dead volume. A syringe pump was used to infuse two different solutions of 5 µL each, one in each inlet (flowrate: 5 μL/min, Legato210P, KDScientific, USA), according to each experiment and in a 1:1 proportion (Table 1). To attain immediate aggregation and maximize the output time, the optimum final concentration of MgCl_2_ was found to be 0.2 M. This salt concentration was studied for the recommended final concentration of Au-nanoprobes of 2.5 nM^7,52^ (more details in Supplementary Information – Fig. S3). The input optical signal was generated by applying a current of 0.4 A to each LED, which was the value that offered the best signal-to-noise ratio (SNR) after interpolating experimental data with the Hermite method^53^ (more details in Supplementary Information – Fig. S4). Data acquisition of electrical output signals generated from LED transmitted light started immediately after the micromixing of infused solutions, in a no flow regime. These measurements were acquired within 3 to 5 min after mixing, with a sampling interval of approximately 1 minute. For baseline measurements, 10 mM phosphate buffer pH 8 solution was used as the transparent medium inside the chip. It was selected as a reliable baseline source as all the tested samples are diluted in this solution. This procedure normalizes the system response for the baseline (*R*_s_ = 1). Sample screenings with *R*_s_ > 1 mean that the output voltage was higher for red light, and correspond to non-aggregated results. Likewise, sample screenings with *R*_s_ ≈ 1 or *R*_s_ < 1 mean that the output voltage was similar or higher for green light which is the characteristic output of aggregated results.

**Table 1:** Experimental conditions used in this work. Detailed description on the components used for each case study and respective concentrations.

	1^st^ case study		2^nd^ case study		3^rd^ case study	
	Inlet 1	Inlet 2	Inlet 1	Inlet 2	Inlet 1	Inlet 2
**Positive trial**	[Au-nanoprobes]: 2.5 nM	—	Hybridized [Au-nanoprobes]:5 nM + [Target oligo]: 200 nM (complementary)	[MgCl_2_]: 0.4 M	Hybridized [Au-nanoprobes]: 5 nM + [Target RNA]: 120 ng/µL (complementary)	[MgCl_2_]: 0.4 M
**after mixing (1:1)**	**2.5 nM**		**2.5 nM + 100 nM + 0.2 M**		**2.5 nM + 60 ng/µL + 0.2 M**	
**Negative trial**	[Au-nanoprobes]:5 nM	[MgCl_2_]:0.4 M	Hybridized[Au-nanoprobes]: 5 nM + [Target oligo]: 200 nM (non- complementary)	[MgCl_2_]:0.4 M	Hybridized[Au-nanoprobes]: 5 nM + [Target RNA]: 120 ng/µL (non- complementary)	[MgCl_2_]:0.4 M
**after mixing (1:1)**	**2.5 nM + 0.2 M**		**2.5 nM + 100 nM + 0.2 M**		**2.5 nM + 60 ng/µL + 0.2 M**	

Microchip sensitivity (Δ*R*_s_) was defined as the difference between the detection responses from assays with positive/non-aggregated results (*R*_s_^+^) and negative/aggregated results (*R*_s_^−^):

2 $${\rm{\Delta }}{R}_{s}={R}_{s}^{+}-{R}_{s}^{-}$$

A one-way ANOVA analysis with Tukey’s Multiple Comparison test using Wolfram Mathematica 10.0 (Champaign, IL) was used to validate the results. One-way analysis of variance was used.

### Case studies

Au-nanoparticles of 14-nm in diameter were functionalized with the ssDNA probe that recognizes a unique sequence on the *BCR-ABL1* oncogene - the hallmark of CML, and fully characterized by UV visible spectroscopy, transmission electron microscopy (TEM) and dynamic light scattering (DLS) – see Supplementary Information – Table S5 and Fig. S5. The performance of the Au-nanoprobes used in this study had been previously thoroughly studied^7^. They were able distinguish several *BCR-ABL1* variants to a 100% accuracy and LOD was 15 ng/µL^7^. Here, LOD for RNA directly extracted from the cells without PCR amplification was found at 40 ng/µL despite its low SNR. No experimental outputs overlapping mismatch results were observed for 60 ng/µL of RNA – see Supplementary Information – Fig. S6.

The microfluidic chip was used in conjunction with Au-nanoprobe assays to detect the specific RNA sequence associated with CML in three progressive case studies (Table 1). The first case study was performed to verify the colorimetric screening and reproducibility of hybridized Au-nanoprobes with/without salt (proof-of-concept). For the second case study, synthetic oligonucleotide sequences were added to Au-nanoprobes and mixed with salt to evaluate the output screening and reproducibility according to the tested sequence (complementary/non-complementary sequence of CML). This study was done as an intermediate step towards clinical trials. The only addition to Au-nanoprobes and salt is the synthetic oligonucleotide, either complementary or non-complementary. The complementary oligo sequences show a perfect match to the Au-nanoprobes, thus representing the best-case scenario of a positive match. To mimic clinical trials, the third case study uses RNA extracted from culture cells (K562, positive for BCR-ABL1/THP1, negative control) instead of synthetic DNA sequences, to evaluate the output screening and reproducibility of results. Due the complexity of RNA solutions, positive controls extracted from cells tend to have a lower affinity to Au-nanoprobes when compared to complementary synthetic oligonucleotide sequences but constitute a better comparison to the real clinical sample.

**Figure 3:**
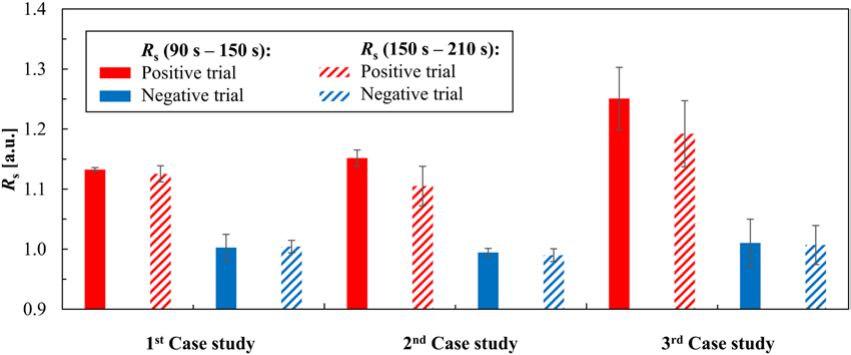
Colorimetric results via proposed microfluidic chip for each case study. Measurements were performed in 90–150 seconds (opaque bars) and 150–210 seconds (patterned bars) after filling the detection chamber. Flow was at rest during data acquisition. Bars represent the average *R*_s_ of independent measurements (n ≥ 3) and error bars indicate standard deviations. Statistical analysis was performed with Mathematica 10.0, using one-way ANOVA and Tukey’s comparison test (p < 0.005).

A discriminated response between complementary (POS) and non-complementary (NEG) assays was obtained for all 3 tested cases (Fig. 3). Performed statistical analysis of variance (ANOVA) with Tukey’s comparison test output strongly suggested that positive and negative results belong to different populations (p <0.005), yielding a significant discrimination between POS and NEG.

**Figure 4:**
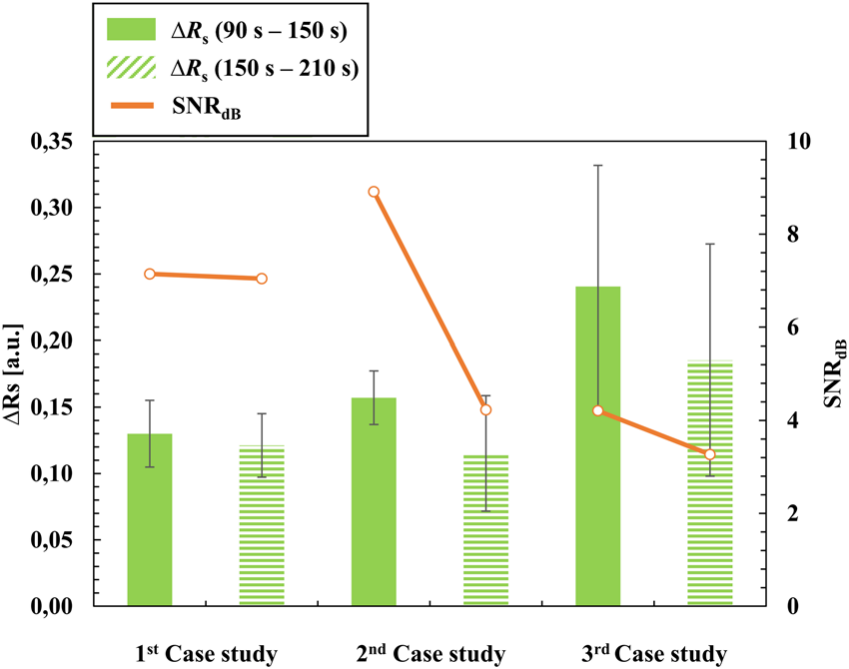
Sensitivity between positive and negative colorimetric results, within 90–150 seconds (opaque bars) and 150–210 seconds (patterned bars), and signal-to-noise ratio trend over time (orange lines) for each case study. Bars represent the average ∆*R*_s_ of independent measurements (n ≥ 3) and the error bars indicate their standard deviations.

For the first case study, the *R*_s_ values obtained showed the least dependency on time. The absence of biological targets and other impurities was a major contributor to this consistency (Fig. 3). The inclusion of synthetic oligonucleotides and RNA, in the second and third case studies respectively, increased the final solution density and added more variability, increasing the measurement errors. Positive measurements in these studies had *R*_s_ decrease over time. The presence of high salt concentration values may have promoted Au-nanoprobe aggregation and precipitation onto the microchannel over time, causing these losses in the ratio. Negative trials showed aggregation occurred sooner than 90 seconds, and thus, all the acquired data in these trials presented a stabilization ratio of *R*_s_ ≈1.00. The *R*_s_ average values increased for the positive trials of the second and third case studies due to an increase in absorption of green light and decrease in absorption of red light, mainly caused by the non-aggregated Au-nanoprobes and respective targets. Experimental results showed that SNR decreases both with time and complexity of experiments, i.e. species in solution. The best SNR was attained between 90 and 150 seconds, providing for the strongest outputs in the shortest frames of time (Fig. 4). The device’s SNR_dB_ was calculated according to:

3 $$SN{R}_{dB}=10Lo{g}_{10}(\frac{{\rm{\Delta }}{R}_{s}}{\delta {\rm{\Delta }}{R}_{s}})$$

where ∆*R*_s_ and δ∆*R*_s_ are the device’s sensitivity and sensitivity error, respectively. Presence of target molecules (synthetic oligonucleotides and/or total RNA) appeared to induce an increase to the detection sensitivity, even though a slight increase to the noise was also perceived.

Time to data output was at least 10 times faster when to the off-chip Au-nanoprobe assay^7^ and 27 times faster when compared to conventional screening techniques for CML (RT-PCR)^38,54^ or to the few existent optical screenings with Au-nanoprobes at microscale^12^. The promotion of rapid kinetics of aggregation of Au-nanoprobes is enhanced by the proposed device, which greatly improves qualitative data output.

## Conclusions

A novel multifunctional microchip was developed and used to detect *BCR-ABL1*, the fusion transcript responsible for Chronic Myeloid Leukemia (CML), directly from total RNA extracted from cells, via colorimetric Au-nanoprobe non-cross-linking assay. The overall design and assembly was implemented taking into account ease of scale up production and maintaining low cost fabrication. These characteristics make the device suitable for a disposable POC platform with a non-disposable host device integrating the two LED light sources, photodetector and electronic circuit with signal amplification, analogue to digital conversion and PC connectivity. Optimization of the mixing device made possible the detection of target RNA in a small sample volume (10 µL), thus reducing the amount of sample required (3 × less than conventional setups^12^).

Volume might be further reduced via addition of a non-miscible buffer (mineral oil) with the sole purpose of pushing the sample solution through the mixing and optical stages, as the colorimetric detection step requires only 50 nL (volume of the filled detection channel)^55,56^. The setup itself can also be optimized towards POC by miniaturizing its components, such as designing and incorporating a mechanical pump to the microfluidic chip^57^ and shortening its optical path length.

The gold standard for CML detection and follow-up is Quantitative Polymerase Chan Reaction (RT-qPCR), with a cost of €20 per sample. Based on current costs for Au-nanoprobe colorimetric assay, the estimated cost is €0.20–1.00 per assay^7^. A microfluidic chip, integrating all steps for diagnostics, will depend on production setup that would bring costs of standalone chip operation to €10, but offering unique advantages: the microfluidic chip presented good sensitivity and was able to yield accurate results in a remarkably short period of time, due to the fast transition between mixing and detection stages provided by the chip’s design, and the process becomes available anywhere due to the possibility of portability. Moreover, the total cost of each assay can be further decreased with mass production of such devices.

Since the colorimetric method for CML screening has been fully validated in mimicked clinical samples, future work shall focus on the integration of this microfluidic platform into a stand-alone device that will open the possibility to more complex, automatized and cost-effective generic DNA/RNA tests suitable for POC screening. In this specific case, it will improve the early diagnosis of CML and provide an easy to use platform for follow up with patients.

## Materials and Methods

### Materials

All chemicals were of molecular biology grade and purchased from Sigma-Aldrich. Ultrapure water used in these processes came from a Millipore water purification system (Merck Millipore, USA). Oligonucleotides were provided by STABVIDA, Portugal. SU-8 2050 photoresist, propylene glycolmethyl ether acetate developer (PGMEA) and customized masks, used during the lithography process of the microchip fabrication, were obtained from Microchem (Microchem, USA) and JDPhoto (JDPhoto-tools, UK), respectively. Epoxy resin ES562, used as a master mold for PDMS soft lithography, was ordered from Permabond (Permabond, UK). For the PDMS microchips fabrication, a Sylgard 184 Silicone Elastomer Kit was used (DowCorning, Spain). To improve separation between the PDMS and SU-8 mold, a silanization step was performed onto the SU-8 mold using tridecafluoro-1,1,2,2-tetrahydrooctyl trichlorosilane (Microchem, USA). Microfluidic connections between chip and reservoirs were made using an optimized Teflon tubing kit, ordered from Elveflow (ElveSys SAS, France).

### Fabrication of the microfluidic chip

Microfluidic chips followed standard fabrication steps^58,59^. A mask with the chip’s pattern was designed in AutoCAD 2014 (AutoDesk, USA). This mask was then printed on sodalime glass using chrome as ink for a precise definition. The first mold, on SU-8, was patterned by ultra-violet photolithography. SU-8 was spin-coated on silicon wafers at 1400 rpm (Karl Suss CT62/ Suss MicroTec, Germany) to form a layer with approximately 125 μm, soft baked on a levelled hotplate at 65 °C during 5 min, followed by 25 min at 95 °C, then left for relaxation and cool down for 10 min. Afterwards, they were exposed on a mask aligner (MA6, SussMicroTec, Germany) for 18 s with an exposure dose of 310 mJ/cm^2^. The designed photolithographic mask was used in this step, together with an i-line filter to expose with the recommended wavelength of 365 nm. A post-bake took place for a duration of 5 min at 65 °C, followed by 11 min at 95 °C. The samples were then submerged to develop in PGMEA for approximately 12 min. A magnetic agitation at 500 rpm was used to enhance this process. In the end, samples were rinsed with isopropanol, and dried with compressed nitrogen.

A replica from the SU-8 mold was made using epoxy resin, which presents higher durability without losing the definition required to fabricate several chips^60,61^. The SU-8 mold was first silanized in a vacuum desiccator to facilitate the posterior removal of PDMS. PDMS was prepared by mixing base and curing agents in a 10:1 weight proportion, stirred, degassed in a vacuum desiccator and poured over the SU-8 mold for the curing process in a levelled oven for 4 hours at 65 °C. The PDMS slab fabricated in this process, with the complementary pattern, was subsequently peeled off from the SU-8 mold and placed on top of a Petri dish with features faced up. On top of the PDMS slab, epoxy resin was poured until a layer of approximately 2 mm in thickness was formed. Another degassing step took place in the vacuum desiccator to remove bubbles trapped in epoxy. To finish this process, epoxy was cured in the levelled oven at 120 °C for 1 hour and peeled from the PDMS. The resulting epoxy mold was then used for soft lithography fabrication of the PDMS chips. This procedure took the same steps as those described above for the PDMS slab.

For the inlets and outlet, PDMS chips were punched using a razor sharp stainless steel biopsy puncher with 1.25 mm outer diameter. The chips were irreversibly bonded to glass slides, using oxygen plasma for 70 s at 98 mTorr with an applied power of 100 W on a Trion Minilock Phantom III RIE (TrionTech, USA). To complete the plasma sealing, PDMS chips already bonded to glass were baked at 100 °C for 5 min to increase the bond strength.

Samples were characterized by optical microscopy (Leitz Laborlux12MEST, Leica, Germany), confocal scanning microscopy (LSM700, CarlZeiss, Germany), profilometry (XP-200, Ambios Technology, Inc., SantaCruz, USA), UV-3101 PC UV/ visible/ NIR double beam spectrophotometry (Shimadzu, Japan) and scanning electron microscopy (SEM-FIB, ZeissAuriga, Germany).

### Optical setup preparation

Two graded-index multimode optical fibers with 62.5 μm core diameter, 125 μm cladding diameter and 0.275 numerical aperture (GIF625, Thorlabs, Germany) were stripped and cleaved from both sides, and inserted in the input and output optical entrances of the chip. One of the two remaining fiber tips was defined as the input fiber and connected to a SMA-ended GIF625 patch cable (Thorlabs, Germany). This cable was either coupled to a high-power green LED (M530F1, dominant wavelength: 530 nm, half width: 33 nm, typical output power: 5.1 mW, Thorlabs, Germany) or red LED (M625F1, dominant wavelength: 625 nm, half width: 18 nm, typical output power: 10.1 mW, Thorlabs, Germany). A constant current of 0.4 A was applied on both LEDs during the experiments. The other fiber tip was defined as the output fiber and was connected to a pigtailed silicon photodiode (FDSP625, Thorlabs, Germany). Connections with optical fibers were done with bare fiber terminators (BFTU, Thorlabs, Germany) and mating sleeves.

A circuit with an operation amplifier (AD549, Analog Devices, USA) was used to enhance the photodiode output current, and convert it to a voltage signal. The photodiode was connected to the op-amp´s inverting input (virtual ground), and a feedback network that incorporated a capacitor (1.5 nF) and a resistor (20 MΩ). The op-amp’s noninverting input was grounded and the op-amp operation ranged from ± 12 V. Data acquisition as well as command sending were done via the computer using a LabView program (LabView 2013, National Instruments, USA) with a NI USB 6008 interface (National Instruments, USA). The same LabView program was used to operate a Legato 210 P syringe pump (KD Scientific, USA), to automatically infuse and withdraw solutions from the chip.

### Au-nanoprobes and target RNA

Au-nanoprobe design and synthesis were performed as previously described^7^. The 14 nm AuNPs prepared by the citrate reduction method were functionalized with a thiolated oligonucleotide in 10 mM phosphate buffer (pH 8.0), 0.1 M NaCl. The resulting Au-nanoprobes were stored in the dark at 4 °C and characterized by UV visible spectroscopy, transmission electron microscopy (TEM) and dynamic light scattering (DLS).

The probe sequence and the complementary target were derived from the *BCR-ABL1* e14a2 (also known as b3a2) chimeric protein mRNA (GeneBank Accession No AJ131466.1), which is the most frequent breakpoint in CML, accounting for 55% of cases^54,62^. Au-nanoprobe selectivity was assessed against two non-modified synthetic oligonucleotides: a complementary target corresponding to the *BCR-ABL1* e14a2 mRNA sequence, and an unrelated sequence was used as non-complementary target (5′-AGA AGA GCT ACG AGC TGC CCG ATG GCC AGG TCA TCA CCAT-3′).

Immortalized human cell lines derived from a CML patient in blast crisis, K562 (*BCR-ABL1* e14a2 fusion transcript positive cell line) and from an acute monocytic leukemia patient, THP1 (*BCR-ABL1* negative) were cultured, respectively, in DMEM and RPMI with 10% (v/v) FBS, at 37 °C with 5% (v/v) CO_2_. These cell lines were used as positive and negative controls for the presence and absence of *BCR-ABL1* transcript, respectively.

Total RNA was extracted from K562 and THP1 cell pellets by the guanidine thiocyanate procedure (SV Total RNA Isolation System, Promega)^7^. RNA was resuspended in DEPC-treated water and stored at −80 °C until use. RNA concentration and purity was determined by UV spectrophotometry.

## Electronic supplementary material

Supplementary Information
