# Supplementary material for: Multifunctional microfluidic chip for optical nanoprobe based RNA detection – application to Chronic Myeloid Leukemia
Source: Sci Rep. 2018 Jan 10;8:381. doi: 10.1038/s41598-017-18725-9 (PMC5762653; doi:10.1038/s41598-017-18725-9)
Supplement: Supplementary file 1 — Supplementary Information [file 41598_2017_18725_MOESM1_ESM.doc]

**Supplementary material**

**Multifunctional microfluidic chip for gold nanoprobe based RNA detection with built-in passive micromixer and optical detection modules – application to *Chronic Myeloid Leukemia***

Pedro Urbano Alves,*^a^* Raquel Vinhas,*^b^* Alexandra R. Fernandes,*^b^* Semra Zuhal Birol,*^c^* Levent Trabzon,*^c^* Iwona Bernacka-Wojcik,*^d^* Rui Igreja,*^a^* Paulo Lopes,^e^ Pedro Viana Baptista,*^*b^* Hugo Águas,^*^*^a^* Rodrigo Martins,*^a^* Elvira Fortunato,*^a^*

^a^CENIMAT/I3N, Departamento de Ciência dos Materiais, Faculdade de Ciências e Tecnologia, Universidade Nova de Lisboa, Campus de Caparica, 2829-516 Caparica, Portugal

^b^UCIBIO, Departamento de Ciências da Vida, Faculdade de Ciências e Tecnologia, Universidade Nova de Lisboa, Campus de Caparica, 2829-516 Caparica, Portugal

^c^MEMS, Department of Nanoscience and Nanoengineering, Istanbul Technical University, Ayazaga Campus 34469 Maslak, Turkey

^d^Laboratory of Organic Electronics, Department of Science and Technology, Linköping University, SE-601 74 Norrköping, Sweden

^e^Department of Physics and IEETA (Institute of Electronics and Informatics Engineering of Aveiro), Campus Santiago, University of Aveiro, Aveiro 3810-193, Portugal

*Corresponding authors:

H. Águas [hma@fct.unl.pt](mailto:hma@fct.unl.pt); P. V. Baptista [pmvb@fct.unl.pt](mailto:pmvb@fct.unl.pt)

Tel.: +351 212948525


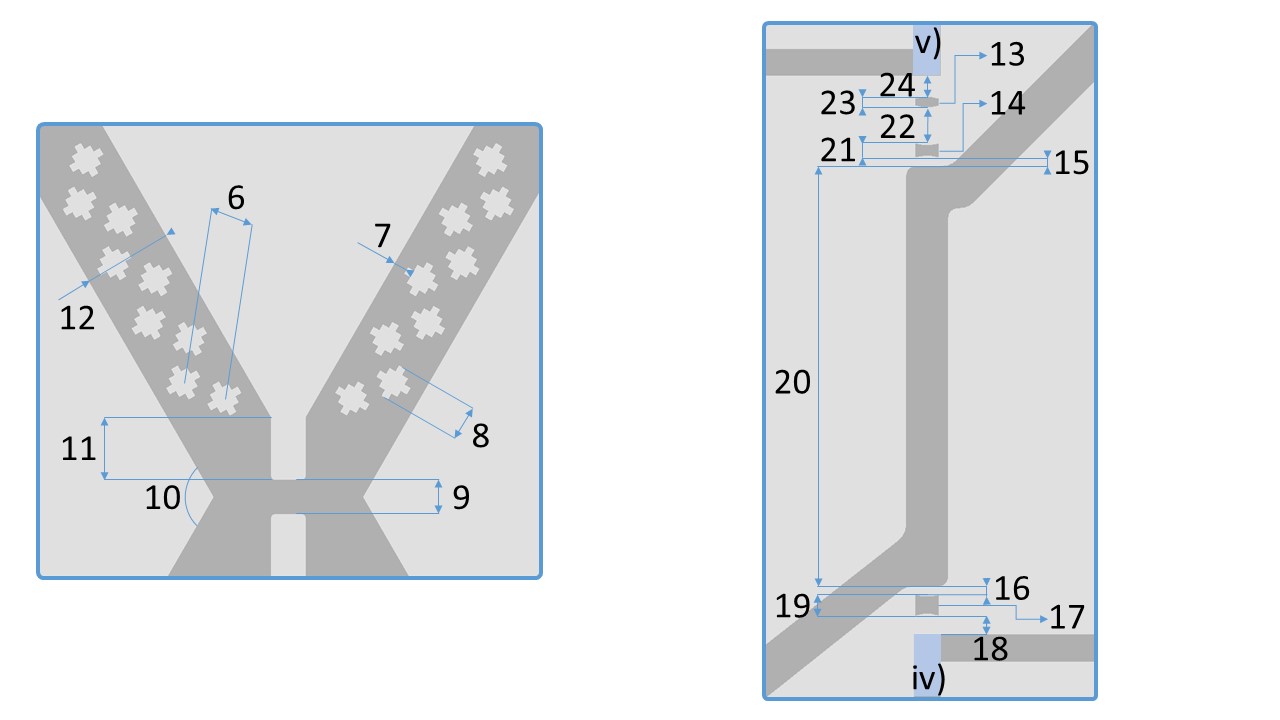


**A**

**B**

**Figure S1.** Microfluidic chip – Mixing and detection design details.

**A** - Mixing microchannel - detailed view. 6- distance between diamond shaped obstacles: 128µm; 7-distance between diamond shaped obstacles and wall: 40µm; 8- diamond shaped obstacle width: 100µm; 9- throttle width: 100µm; 10- Rhombi angle: 120º; 11- Gap between throttle and channel: 12-180µm; mixer channel width: 260µm.

**B** - Optical detection microchannel – detailed view. 13- 3^rd^ lens curvature radius/width: 180µm/110µm; 14- 2^nd^ lens curvature radius/width: 210µm/110µm; 15- min. distance between 2^nd^ lens and channel: 42.5µm; 16- min. distance between 1^st^ lens and channel: 41µm; 17- 1^st^ lens curvature radius/width: 175µm/110µm; 18- min. distance between 1^st^ lens and optical fiber: 91µm; 19- 1^st^ lens max. thickness: 98µm; 20- Optical path length inside the channel: 2mm; 21- 2^nd^ lens max. thickness: 65µm; 22- min. distance between 2^nd^ and 3^rd^ lenses: 173µm; 23-3^rd^ lens max. thickness: 50µm; 24- distance between 3^rd^ lens and optical fiber: 100µm. iv) and v) mark the spot of input and output optical fibers, respectively, with a groove width of 127µm.


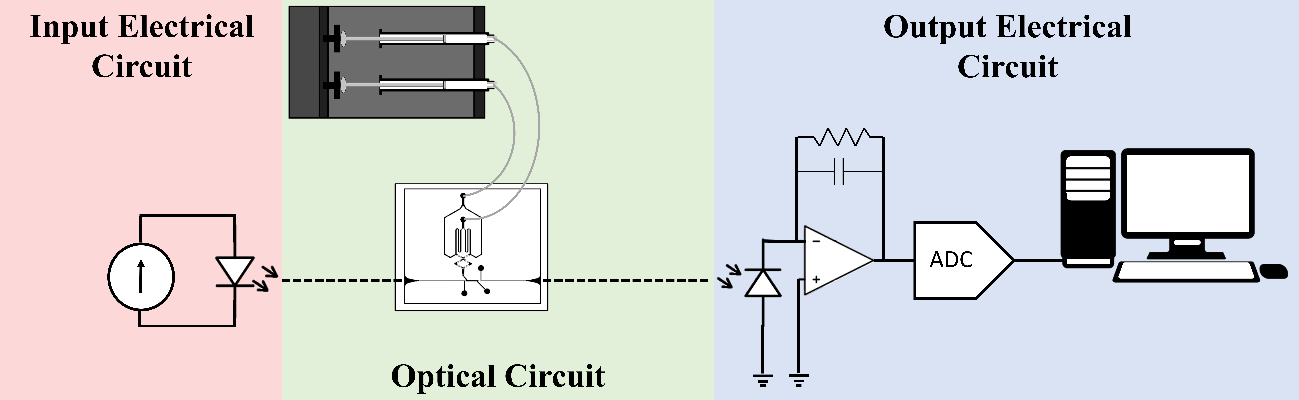


**Figure S2.** Optoelectronic setup scheme: Input electrical circuit – Light with one of two wavelengths (625nm; 530nm) is emitted from a powered LED through an optical fiber, represented by the dashed line; Optical circuit – The microfluidic chip is crossed by 2 optical fiber segments. The first allows incoming light to interact with solutions present in the region where optical detection occurs. The second carries the transmitted light outside the chip. A syringe pump is used to infuse these solutions inside the chip; Output electrical circuit – A photodetector transduces outcoming light to a current signal, which is amplified by an operation amplifier with a feedback network (capacitor: 1.5 nF; resistance: 20 MΩ), and converted to a voltage signal. This analog signal is then converted to digital and acquired by the computer.


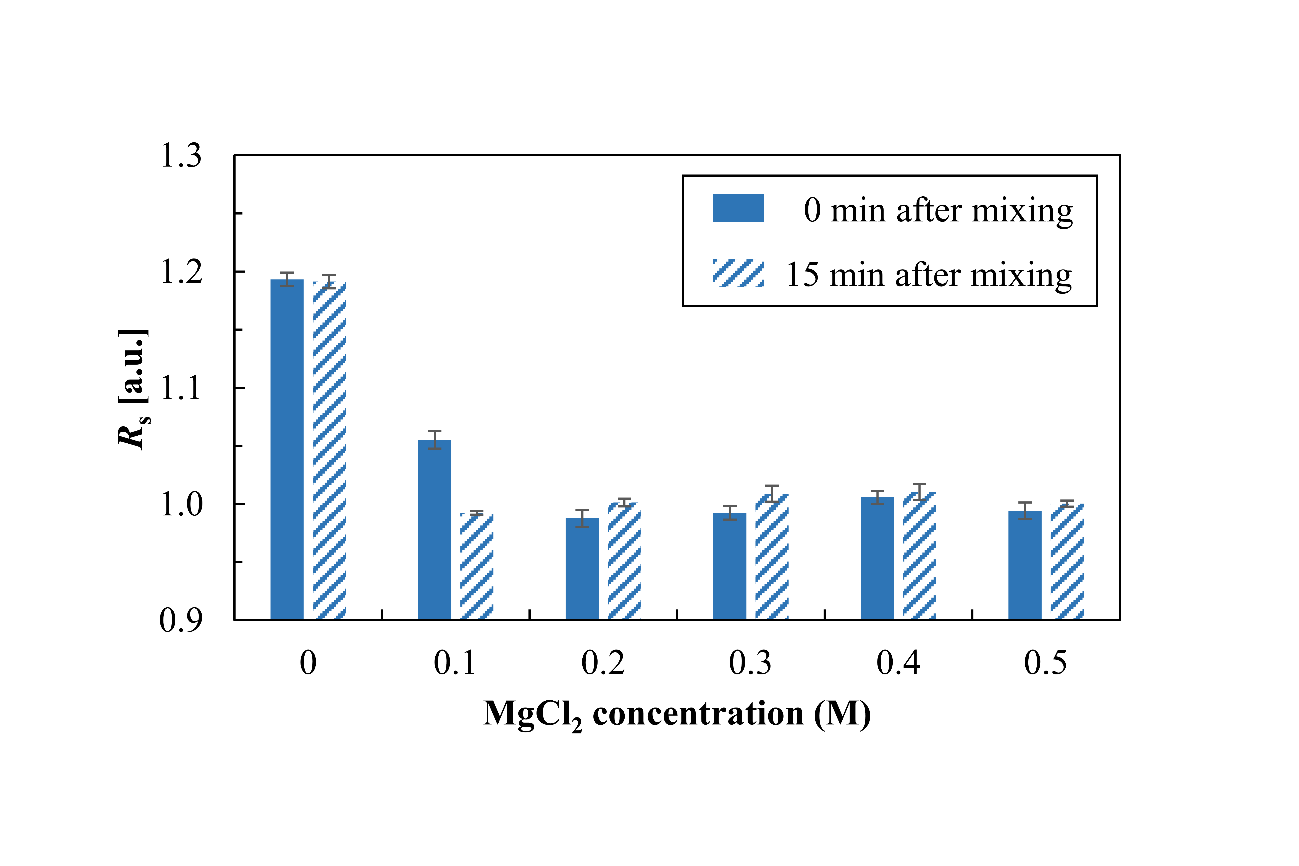


**Figure S3.** MgCl_2_ saturation study for Au-nanoprobes. Ratio measurements performed immediately after mixing and repeated 15 minutes later. kinetic aggregation behavior inside the mixer was studied by mixing 5 nM of Au-nanoprobes and salt solutions with concentrations ranging from 0 to 1 M (1:1). Here, the mixed solution was studied for an Au-nanoprobe final concentration of 2.5 nM, which is the optimum value for RNA detection recommended in literature (Reference 7 of the manuscript). It was optically analyzed as soon as the mixing process finished, and 15 minutes thereafter. Data showed that for salt concentrations of 0.1 M, the complete aggregation state of Au-nanoprobes could only be measured after 15 minutes of mixing. To cause immediate aggregation, at least 0.2 M of salt were needed.


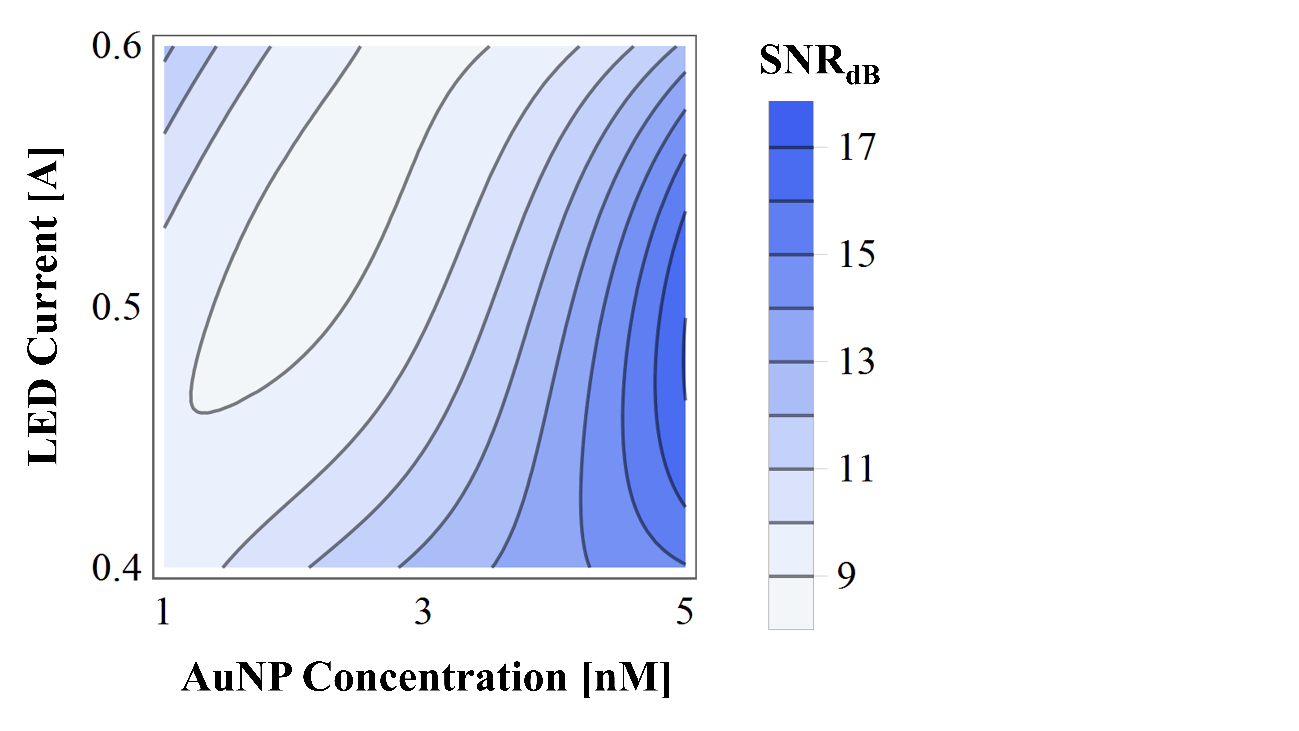


**Figure S4.** Signal-to-noise ratio (SNR) of the device, as a function of LED applied current and Au-nanoprobe concentration of the infused solution. Kinetic aggregation behavior inside the mixer was studied by mixing 5 nM of Au-nanoprobes and salt solutions with concentrations ranging from 0 to 1 M (1:1). Data points were interpolated to find an approximate SNR function within the ranges studied, recurring to the Hermite method. Calculated in Mathematica 10.0.

Although higher concentrations present greater sensitivity, the optimum value for RNA detection recommended in literature (reference 7 of the manuscript) is 2.5 nM. This concentration was therefore used to read the highest SNR_dB_ value, namely 0.4 A. Subsequent measurements were made by applying 0.4 A to both LEDs.

**Table S5**. AuNPs and Au-nanoprobe characterization through Transmission Electron Microscopy (TEM), Dynamic Light Scattering (DLS) and UV-Vis.

|  | Au-core average diameter (nm) | Z-average ± SD  (nm) | SPR^1^ peak  (nm) |
| --- | --- | --- | --- |
| AuNPs | 14 | 17.4 ± 0.5 | 519 |
| Au-Nanoprobe | 14 | 26.3 ± 2.0 | 524 |

^1^ SPR – Surface plasmon resonance

**Figure S5**. TEM image of 14 nm citrate capped AuNPs.

**
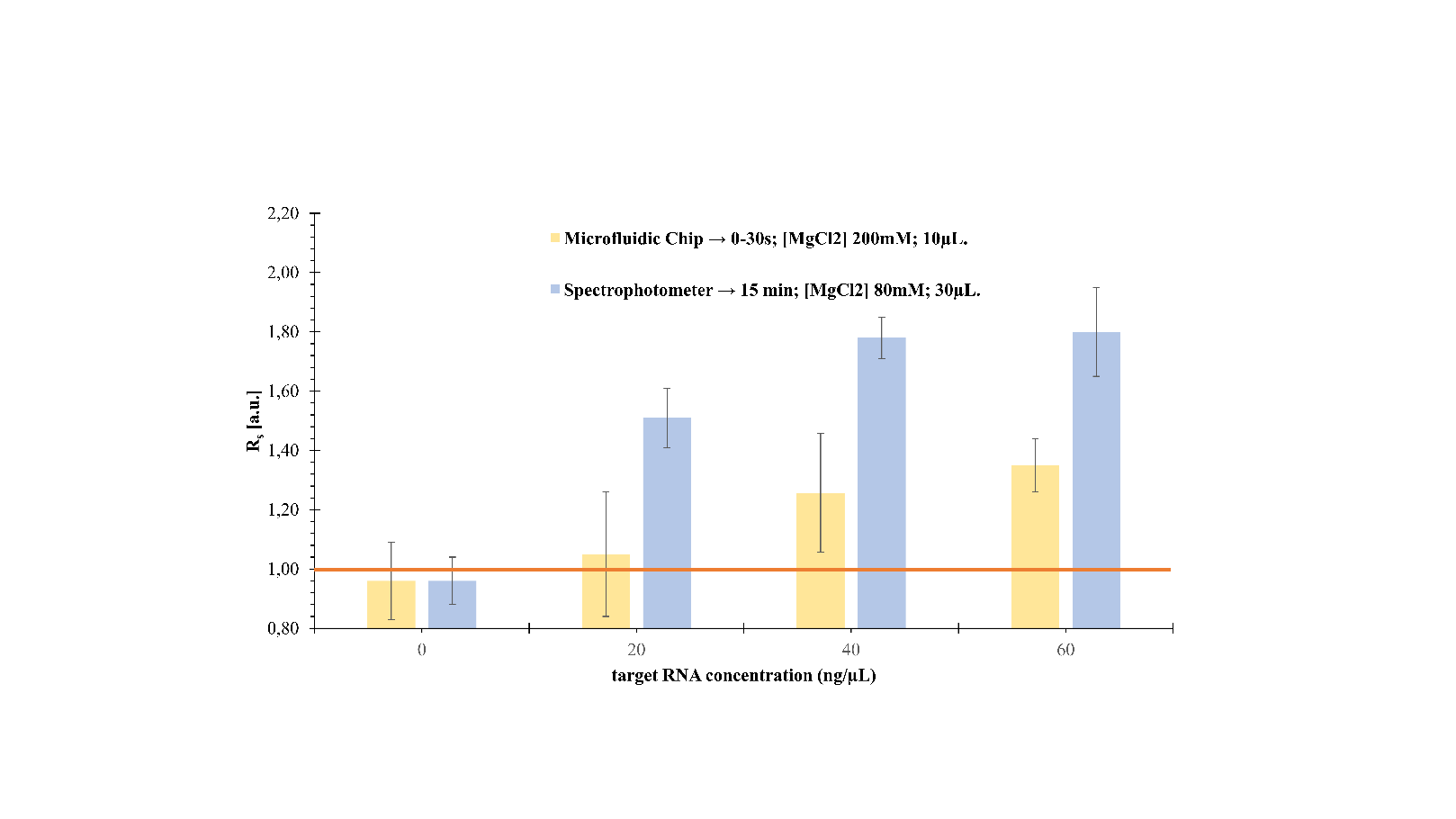
**

**Figure S6.** Limit of detection of *BCR-ABL1* using the corresponding Au-nanoprobes in the designed optical setup with the microfluidic chip, and same limit of detection in a commercial microplate reader (Tecan Infinite® M200 microplate reader - Männedorf, Switzerland). The designed setup consumed 10 µL of total solution in a microfluidic chip, used 0.2 M of MgCl_2_ and performed the screenings at real time. The commercial microplate reader consumed 30 µL of total solution in an Eppendorf, used 80 mM of MgCl_2_ and provided the results 15 minutes after the screening.
